# Supplementary figures and images for: Glucose fluctuation promotes mitochondrial dysfunctions in the cardiomyocyte cell line HL-1
Source: PLoS One. 2023 Sep 21;18(9):e0289475. doi: 10.1371/journal.pone.0289475 (PMC10513336; doi:10.1371/journal.pone.0289475)

## Slide 1
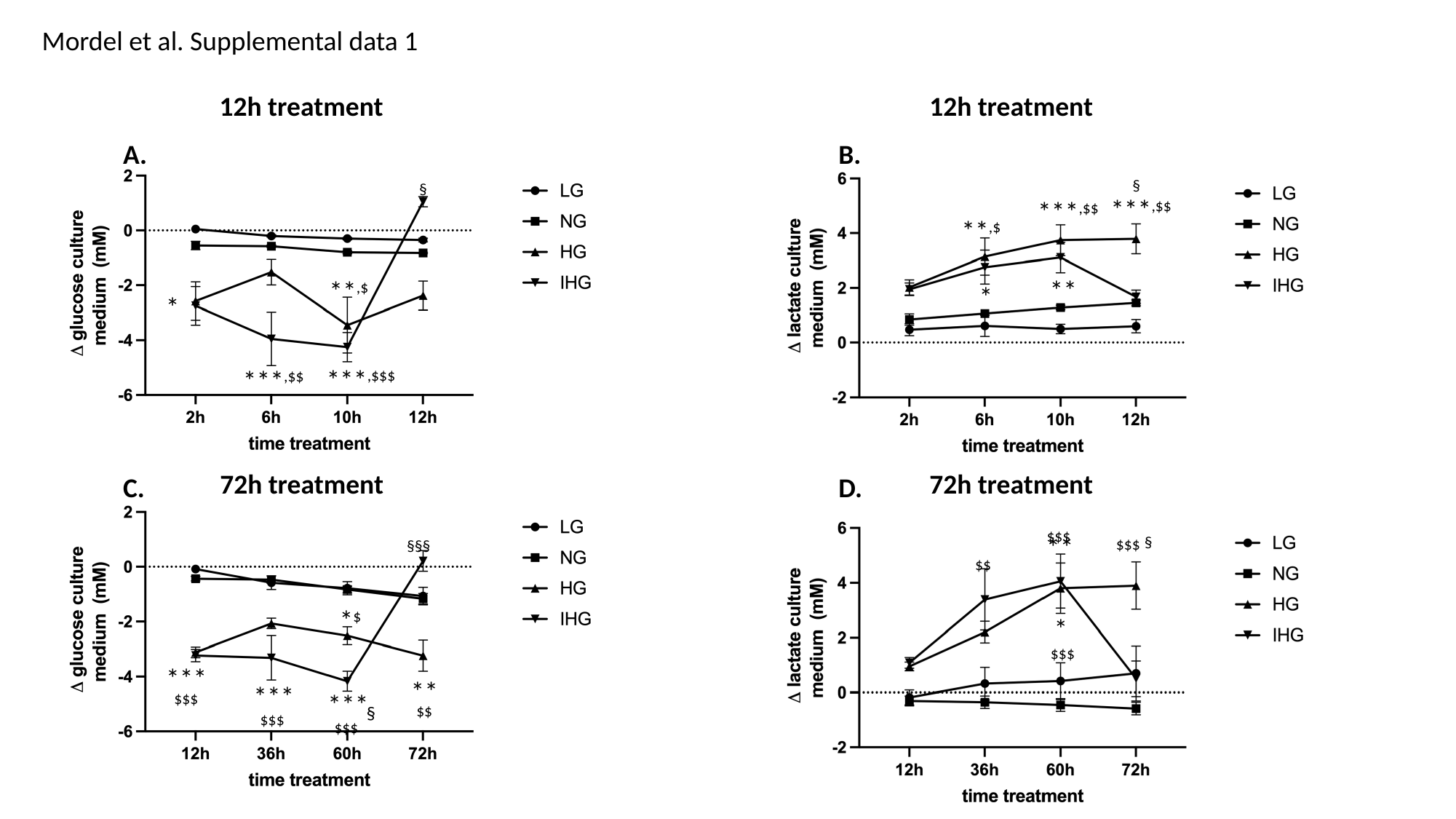

Mordel et al. Supplemental data 1
12h treatment
12h treatment
A.
B.
§
§
***,$$
***,$$
**,$
**
**,$
*
*
***,$$$
***,$$
72h treatment
72h treatment
C.
D.
$$$
§
**
$$$
§§§
$$
*$
*
$$$
***
**
***
$$$
***
$$
§
$$$
$$$

Supplement: S1 Fig — HL-1 cells were cultured at least during 3 weeks with normal glucose (5.5 mmol/l) then submitted to LG, NG, HG or IHG for 12 or 72h. Glucose (A and C) and lactate (B and D) concentrations were measured in the culture medium at different times (see Fig 1) to calculate glucose consumption and lactate production. Data are the means ± S.E.M of 4–6 independent experiments. Two-way ANOVA followed by the Tukey’s multiple comparisons test when evaluating the effect of glucose and time treatment. *, P < 0.05, **, P < 0.01, ***, P < 0.001, vs LG; $, P < 0.05, $ $, P < 0.01, $ $ $, P < 0.001 vs NG; §, P < 0.05, §§§, P < 0.001 vs HG. For 12h treatment and glucose consumption, Ftreatment (3, 48) = 23.85, P<0.0001; Ftime (3, 48) = 7.03, P = 0.0005; FtreatmentXtime (9, 48) = 6.92, P<0.0001. For 12h treatment and lactate production, Ftreatment (3, 48) = 38.90, P<0.0001; Ftime (3, 48) = 3.43, P = 0.0242; FtreatmentXtime (9, 48) = 1.63, P = 0.132. For 72h treatment and glucose consumption, Ftreatment (3, 79) = 49.66, P<0.001; Ftime (3, 79) = 3.602, P = 0.017; FtreatmentXtime (9, 79) = 12.26, P<0.001. For 72h treatment and lactate production, Ftreatment (3, 79) = 22.69, P<0.001; Ftime (3, 79) = 4.308, P = 0.007; FtreatmentXtime (9, 79) = 2.819, P = 0.006. (PPTX) [file pone.0289475.s001.pptx]

## Slide 1
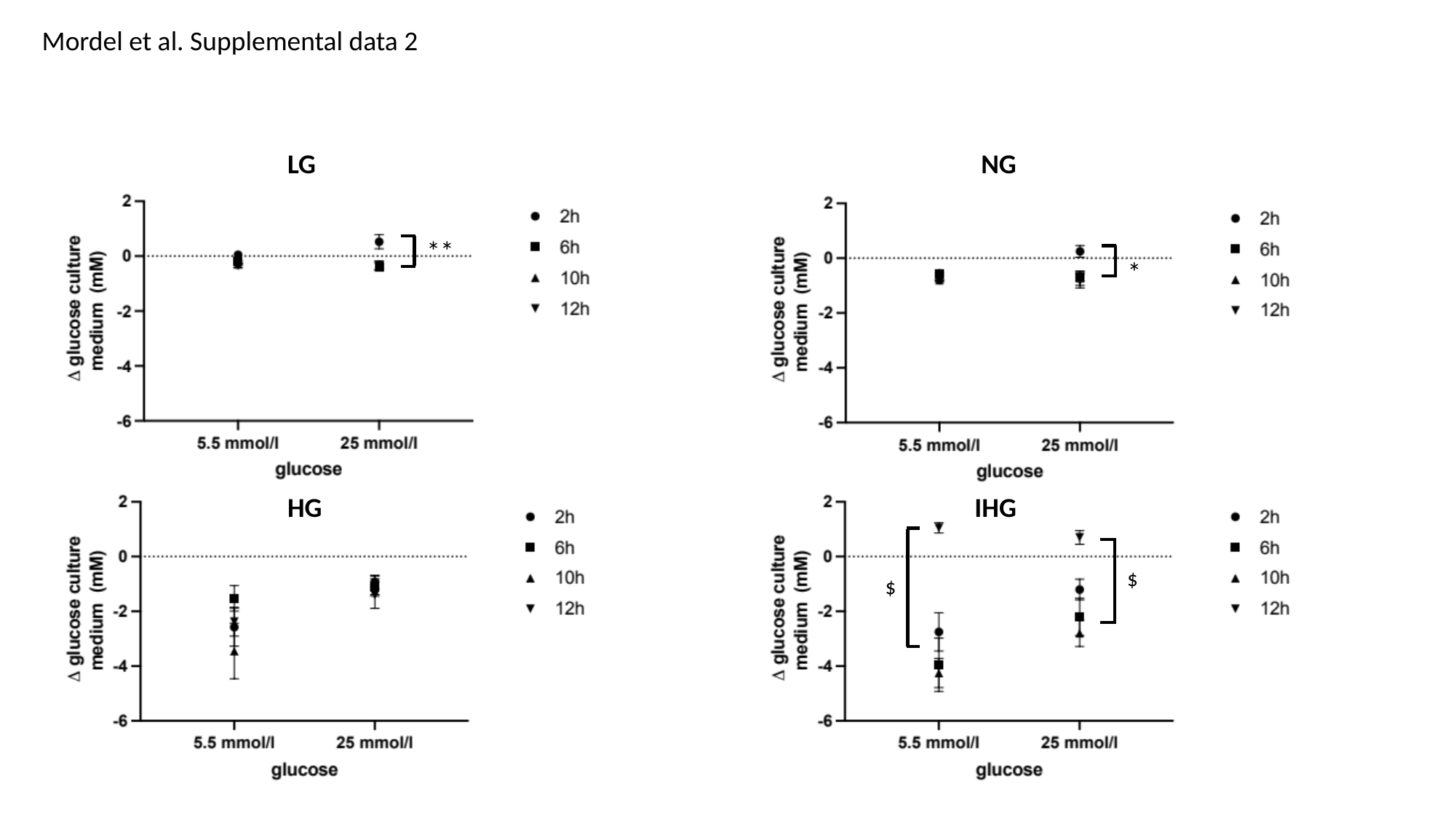

Mordel et al. Supplemental data 2
LG
NG
**
*
HG
IHG
$
$

Supplement: S2 Fig — HL-1 cells were cultured at least during 3 weeks either with normal (5.5 mmol/l) or high (25 mmol/l) glucose then submitted to 4 different regimens for 12h: LG, NG, HG or IHG. Glucose concentration was measured in the culture medium at 0, 2, 6, 10 and 12h to calculate glucose consumption. Data are the means ± S.E.M of 4 independent experiments. Two-way ANOVA followed by the Tukey’s multiple comparisons test when evaluating the effect of time treatment in HL-1 cells culture in normal or high glucose. *, P < 0.05, **, P < 0.01, vs 2h treatment, $, P < 0.05, vs 12h treatment. For LG treatment, Ftreatment (3, 24) = 10.31, P = 0.0002; Ftime (1, 24) = 0.4323, P = 0.5171; FtreatmentXtime (3, 24) = 2.24, P = 0.1093. For NG treatment, Ftreatment (1, 24) = 2.54, P = 0.1241; Ftime (3, 24) = 6.31, P = 0.0026; FtreatmentXtime (3, 24) = 2.85, P = 0.0586. For HG treatment, Ftreatment (1, 24) = 11.65, P = 0.0023; Ftime (3, 24) = 0.974, P = 0.4212; FtreatmentXtime (3, 24) = 1.12, P = 0.3578. For IHG treatment, Ftreatment (1, 24) = 7.31, P = 0.0124; Ftime (3, 24) = 23.63, P<0.0001; FtreatmentXtime (3, 24) = 1.43, P = 0.2570. (PPTX) [file pone.0289475.s002.pptx]

## Slide 1
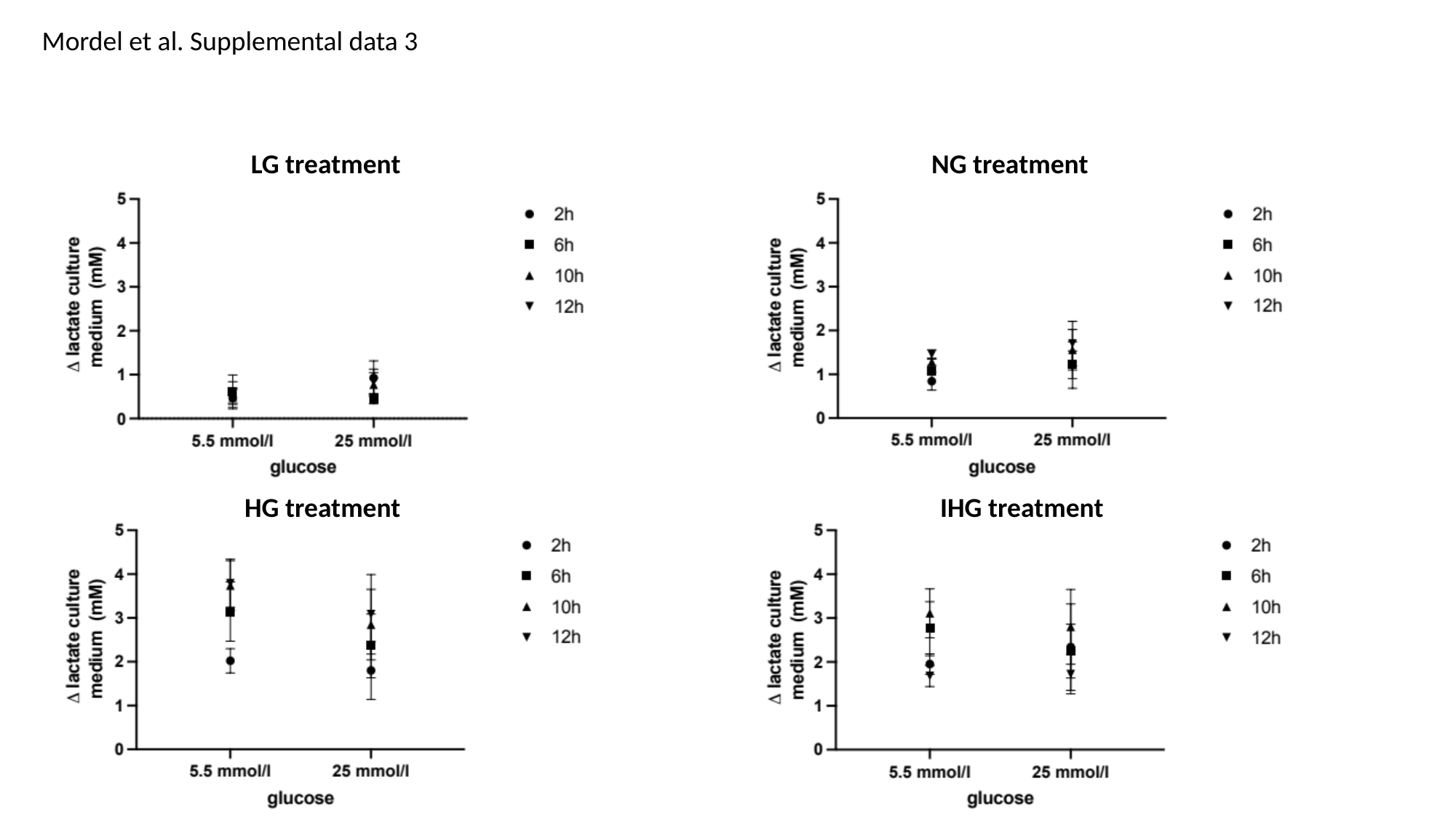

Mordel et al. Supplemental data 3
LG treatment
NG treatment
HG treatment
IHG treatment

Supplement: S3 Fig — HL-1 cells were cultured at least during 3 weeks either with normal (5.5 mmol/l) or high (25 mmol/l) glucose then submitted to 4 different regimens for 12h: LG, NG, HG or IHG. Lactate concentration was measured in the culture medium at 0, 2, 6, 10 and 12h to calculate lactate production. Data are the means ± S.E.M of 4 independent experiments. Two-way ANOVA followed by the Tukey’s multiple comparisons test when evaluating the effect of time treatment in HL-1 cells culture in normal or high glucose. For LG treatment, Ftreatment (1, 24) = 1.09, P = 0.3064; Ftime (3, 24) = 0.18, P = 0.9057; FtreatmentXtime (3, 24) = 0.43, P = 0.7270. For NG treatment, Ftreatment (1, 24) = 1.17, P = 0.2883; Ftime (3, 24) = 1.09, P = 0.3688; FtreatmentXtime (3, 24) = 0.03, P = 0.9914. For HG treatment, Ftreatment "F (1, 24) = 1.89, P = 0.1814; Ftime (3, 24) = 2.13, P = 0.1222; FtreatmentXtime (3, 24) = 0.09, P = 0.9605. For IHG treatment, Ftreatment (1, 24) = 0.05, P = 0.8237; Ftime (3, 24) = 1.47, P = 0.2467; FtreatmentXtime (3, 24) = 0.20, P = 0.8929. (PPTX) [file pone.0289475.s003.pptx]

## Slide 1
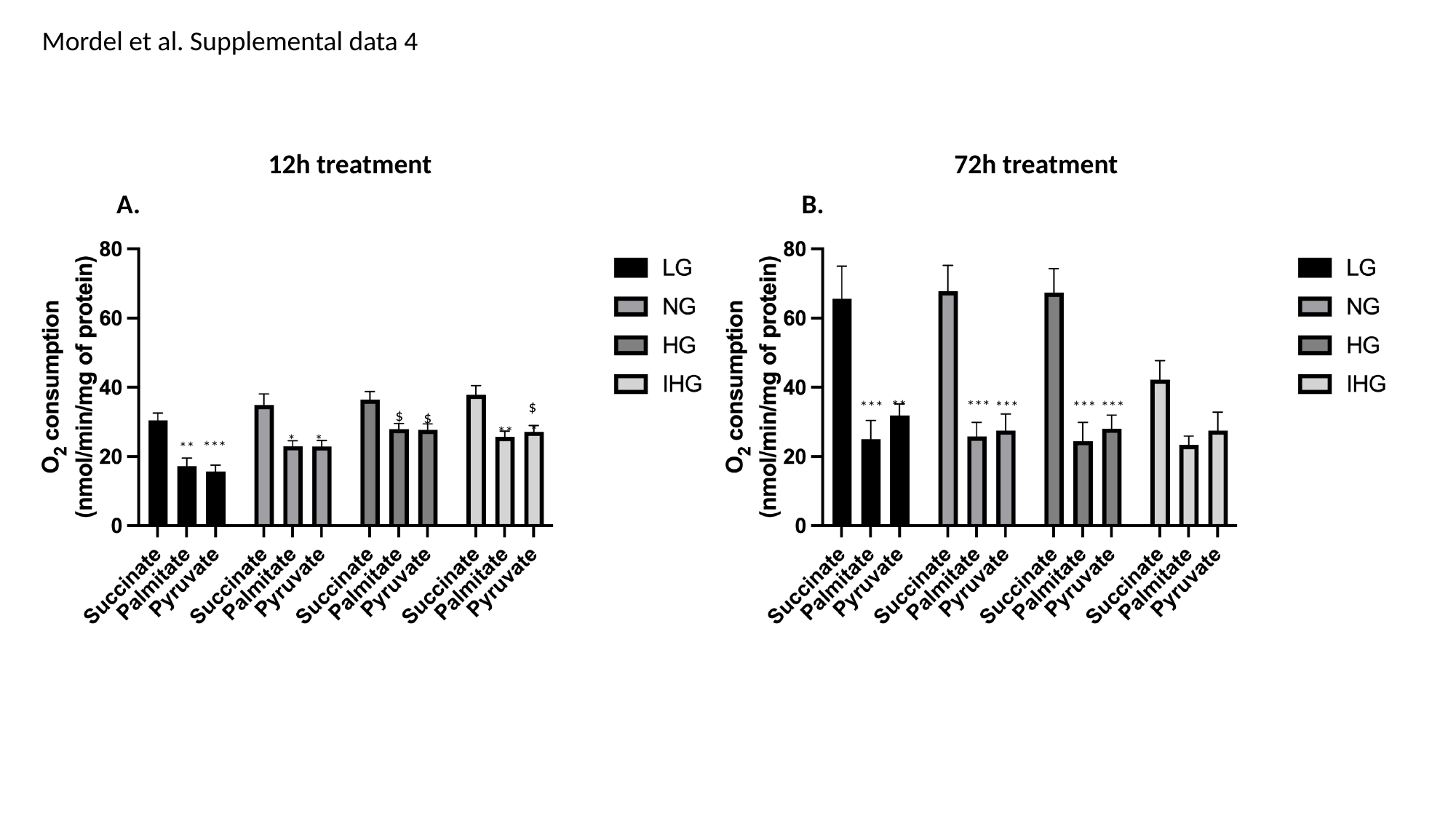

Mordel et al. Supplemental data 4
12h treatment
72h treatment
A.
B.
**
***
***
***
***
***
$
$
$
*
**
*
*
***
**

Supplement: S4 Fig — HL-1 cells were cultured at least during 3 weeks with normal (5.5 mmol/l) glucose then submitted to 4 different regimens (LG, NG, HG or IHG) either for 12 (A) or 72h (B). Oxygen consumption was measured by polarography using 3 different substrates. Data are the means ± S.E.M of 5–7 independent experiments. Two-way ANOVA followed by the Tukey’s multiple comparisons test when evaluating the effect of substrates or glucose treatments. *, P < 0.05, **, P < 0.01, ***, P < 0.001 vs succinate, $, P < 0.05, vs LG treatment. For 12h treatment, Fsubstrate (2, 56) = 39.66, P<0.0001; Ftreatment (3, 56) = 13.15, P<0.0001; FsubstrateXtreatment (6, 56) = 0.453, P = 0.8394. For 72h treatment, Fsubstrate (2, 48) = 48.59, P<0.0001; Ftreatment (3, 48) = 2.038, P = 0.1210; FsubstrateXtreatment (6, 48) = 1.443, P = 0.2182. (PPTX) [file pone.0289475.s004.pptx]

## Slide 1
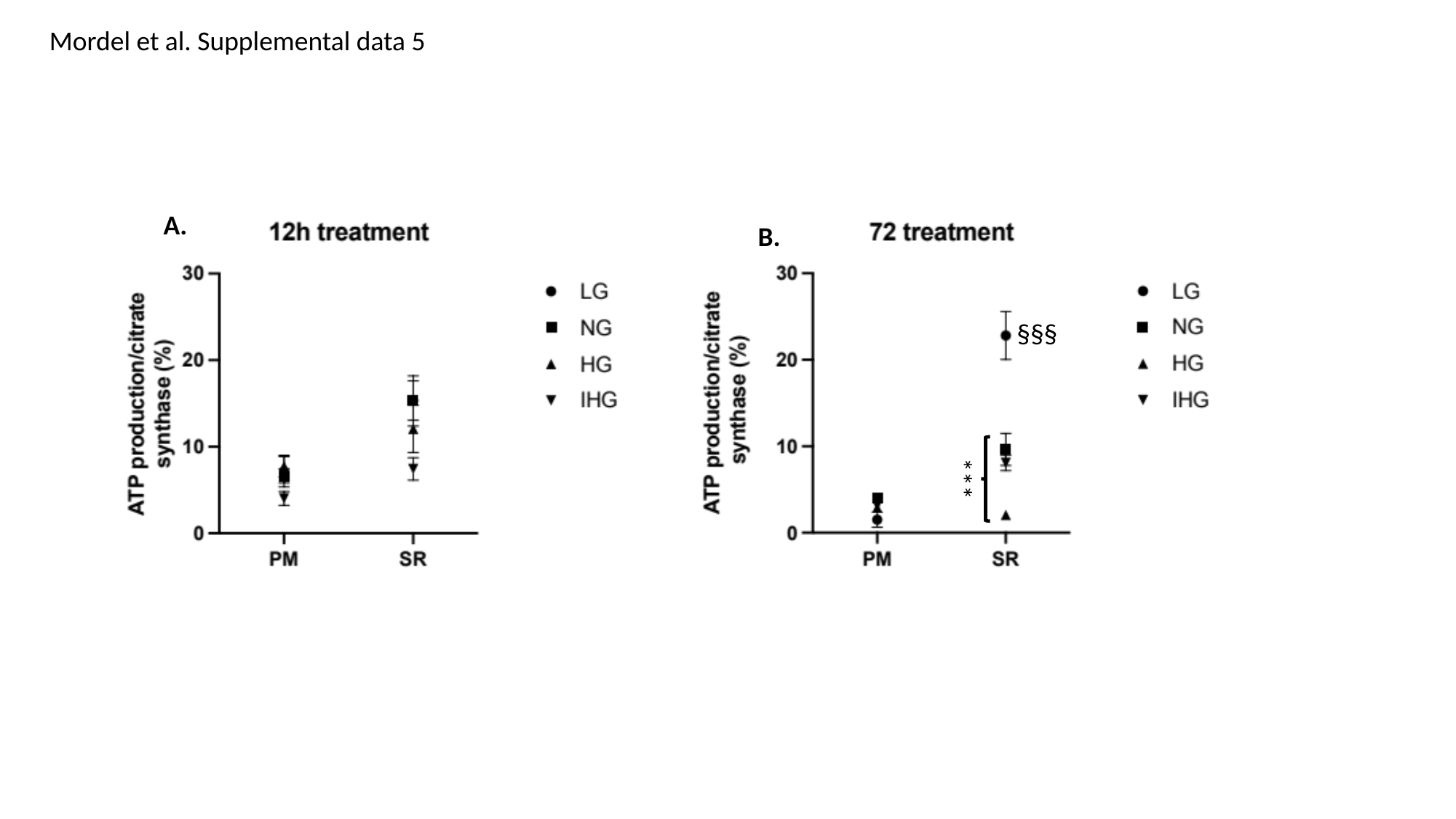

Mordel et al. Supplemental data 5
A.
B.
§§§
***

Supplement: S5 Fig — HL-1 cells were cultured at least during 3 weeks with normal (5.5 mmol/l) glucose then submitted to 4 different regimens (LG, NG, HG or IHG) either for 12 (A) or 72h (B). The mitochondrial ATP production was measured under basal (no substrate) or stimulated conditions (pyruvate + malate or succinate + rotenone). After subtracting ATP level obtained without stimulation, results were normalized to the activity of the citrate synthase activity and expressed in %. Data are the means ± S.E.M of 3–5 independent experiments. Two-way ANOVA followed by the Tukey’s multiple comparisons test when evaluating the effect of substrates or glucose treatments. ***, P < 0.001 vs LG treatment, §§§, P < 0.001 pyruvate + malate vs succinate + rotenone. For 12h treatment, Fsubstrate (1, 22) = 21.000, P = 0.001; Ftreatment (3, 22) = 3.776, P = 0.025; FsubstrateXtreatment (3, 22) = 0.984, P = 0.418. For 72h treatment, Fsubstrate (1, 26) = 57.910, P<0.001; Ftreatment (3, 26) = 15.880, P<0.0001; FsubstrateXtreatment (3, 26) = 22.200, P<0.001. (PPTX) [file pone.0289475.s005.pptx]

## Slide 1
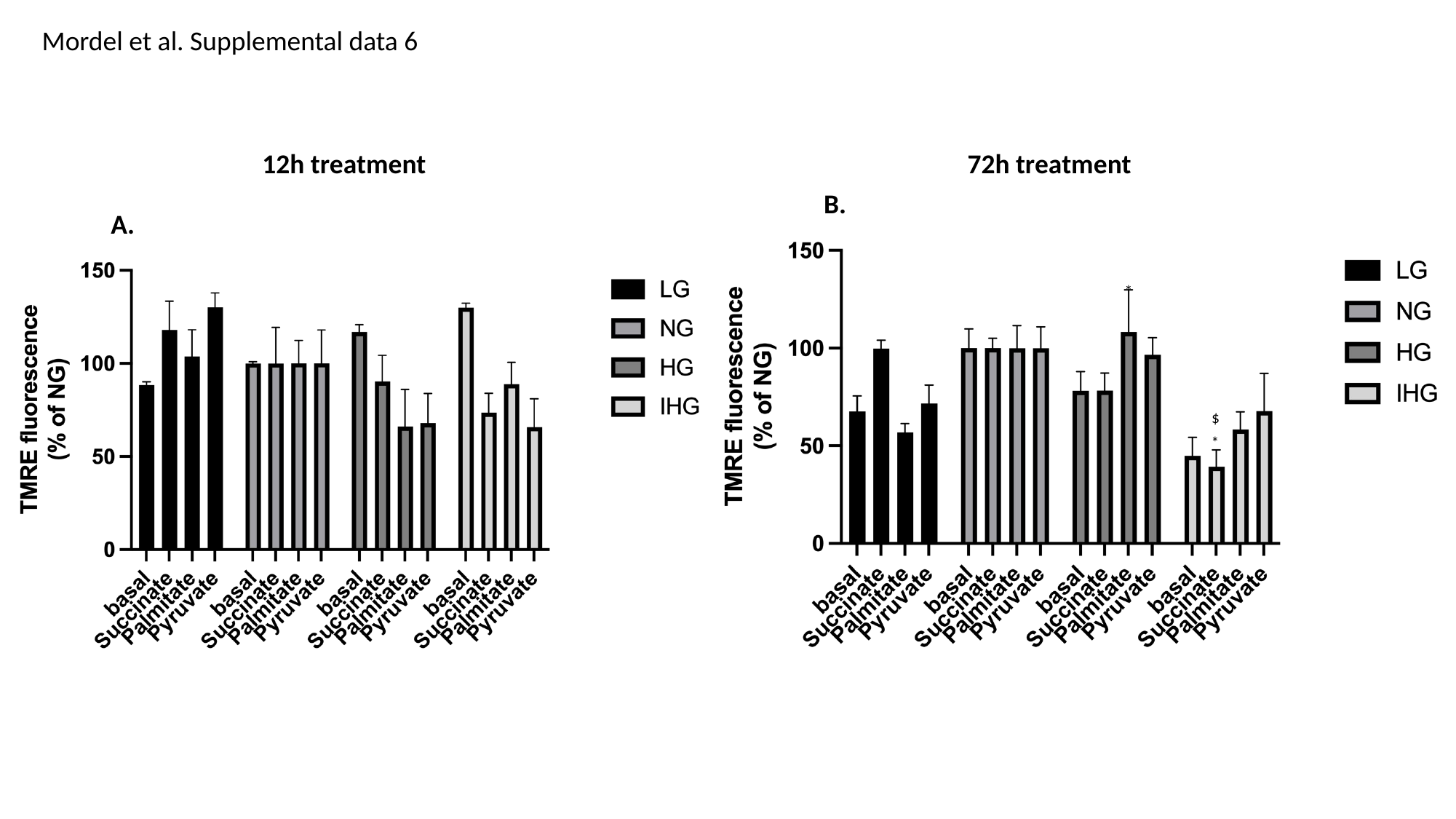

Mordel et al. Supplemental data 6
12h treatment
72h treatment
B.
A.
*
$
*

Supplement: S6 Fig — HL-1 cells were cultured at least during 3 weeks with normal (5.5 mmol/l) glucose then submitted to 4 different regimens (LG, NG, HG or IHG) either for 12 (A) or 72h (B). The MMP was measured using TMRE under basal or stimulated conditions (succinate, palmitate or pyruvate). Results were normalized to the NG condition. Data are the means ± S.E.M of 3–7 independent experiments. Two-way ANOVA followed by the Tukey’s multiple comparisons test when evaluating the effect of substrates or glucose treatments. *, P < 0.05 vs LG treatment, $, P < 0.05, vs NG treatment. For 12h treatment, Fsubstrate (3, 56) = 1.107, P = 0.3541; Ftreatment (3, 56) = 2.212, P = 0.0967; FsubstrateXtreatment (9, 56) = 1.799, P = 0.0887. For 72h treatment, Fsubstrate (3, 64) = 0.5330, P = 0.6613; Ftreatment (3, 64) = 11.53, P<0.0001; FsubstrateXtreatment (9, 64) = 1.619, P = 0.1286. (PPTX) [file pone.0289475.s006.pptx]

## Slide 1
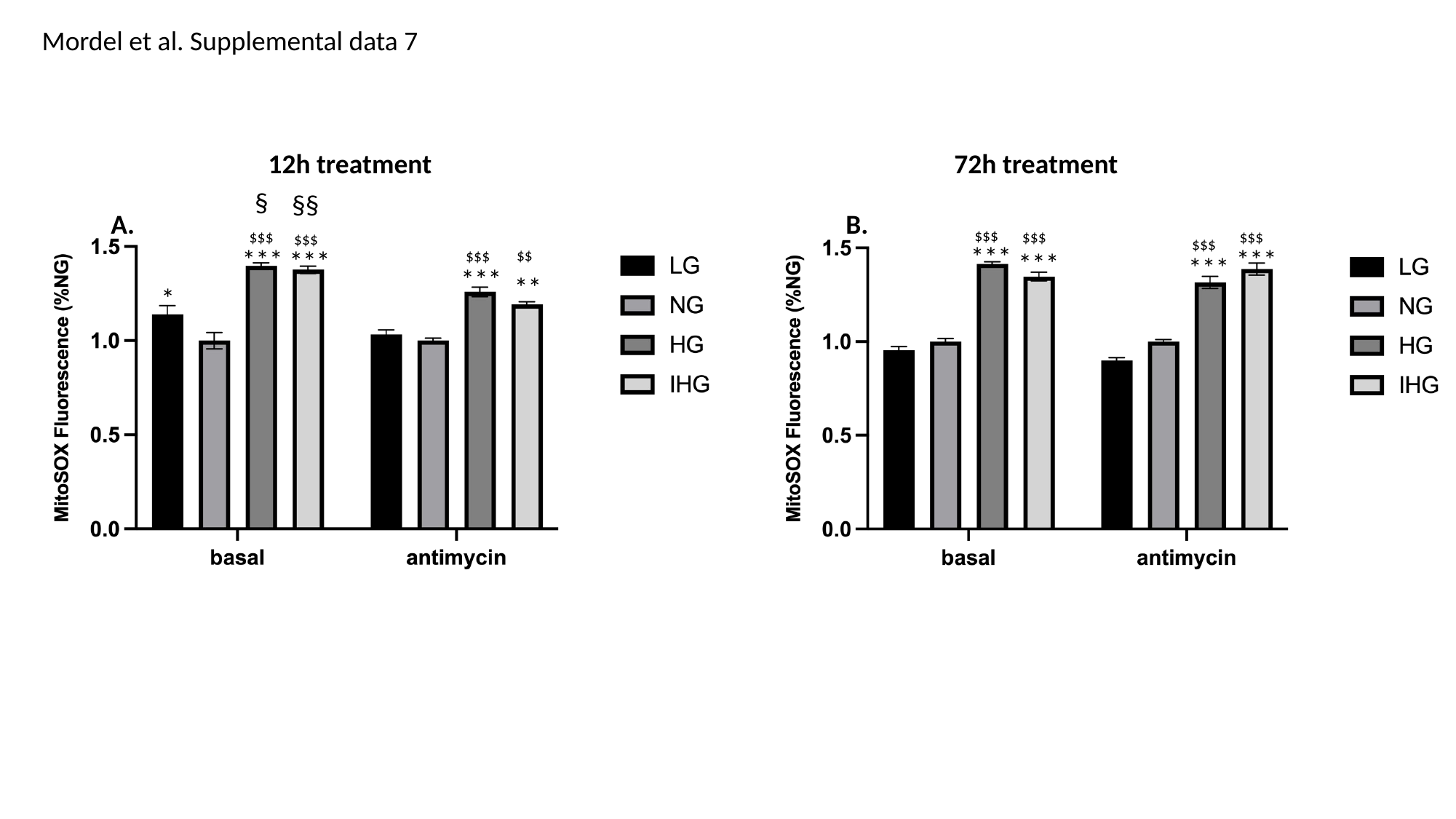

Mordel et al. Supplemental data 7
12h treatment
72h treatment
§
§§
A.
B.
$$$
$$$
$$$
$$$
$$$
$$$
***
$$
***
$$$
***
***
***
***
***
**
*

Supplement: S7 Fig — HL-1 cells were cultured at least during 3 weeks with normal (5.5 mmol/l) glucose then submitted to 4 different regimens (LG, NG, HG or IHG) either for 12 (A) or 72h (B). The mitochondrial superoxide anion production was measured using MitoSox under basal or in the presence of the specific complex III inhibitor, antimycin A. Results were normalized to the NG condition. Data are the means ± S.E.M of 3–4 independent experiments. Two-way ANOVA followed by the Tukey’s multiple comparisons test when evaluating the effect of glucose treatments and antimycin A. *, P < 0.05, **, P < 0.01, ***, P < 0.001 vs LG treatment, $ $, P < 0.01, $ $ $, P < 0.001 vs NG treatment; §, P < 0.05, §§, P < 0.01, basal vs antimycin. For 12h treatment, Fantimycin A (1, 24) = 29.42, P<0.0001; Ftreatment (3, 24) = 63.82, P<0.0001; Fantimycin AXtreatment (3, 24) = 3.914, P = 0.0208. For 72h treatment, Fantimycin A (1, 16) = 3.569, P = 0.0771; Ftreatment (3, 16) = 239.6, P<0.0001; Fantimycin AXtreatment (3, 16) = 4.047, P = 0.0256. (PPTX) [file pone.0289475.s007.pptx]

## Slide 1
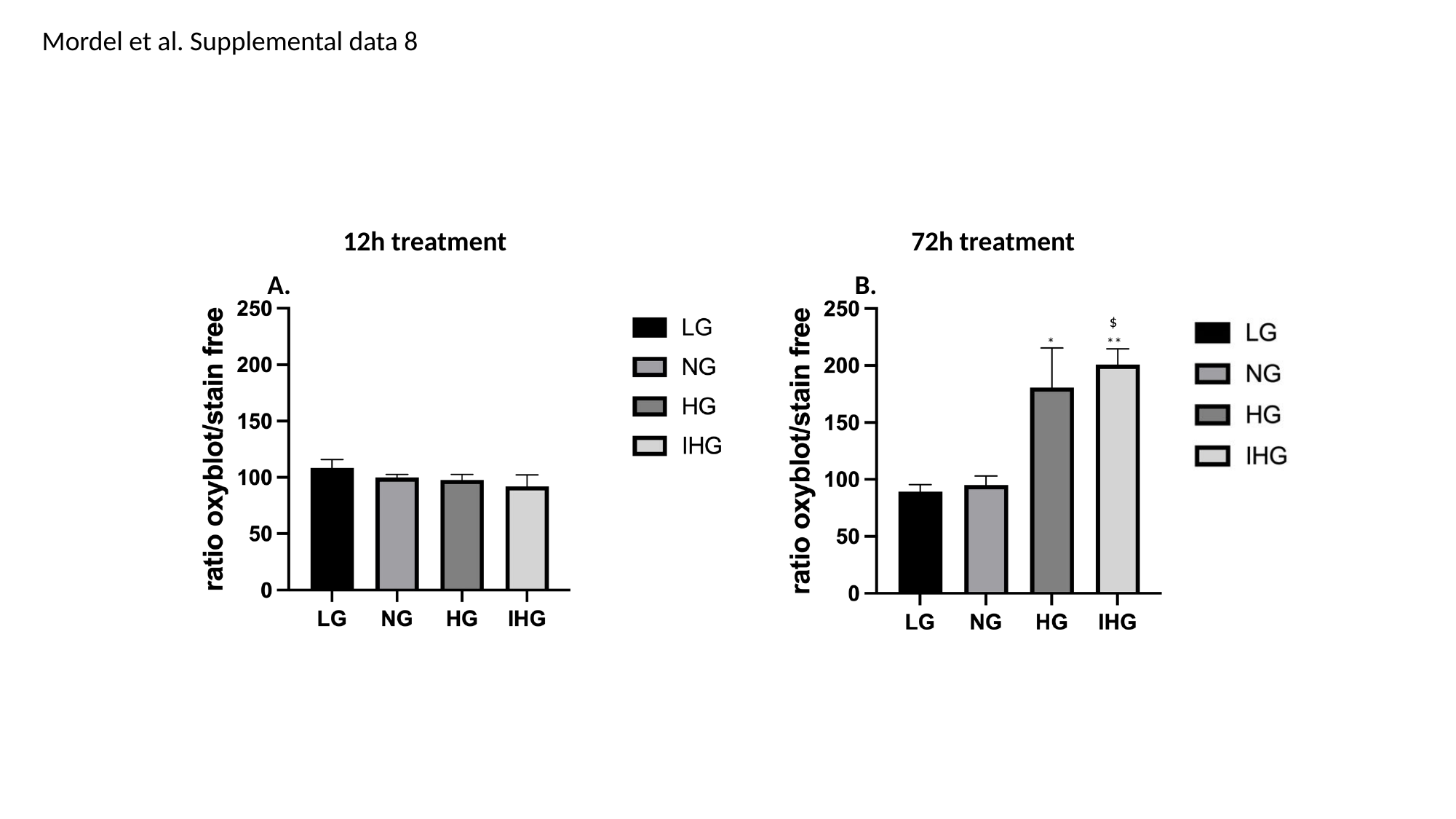

Mordel et al. Supplemental data 8
12h treatment
72h treatment
A.
B.
$
*
**

Supplement: S8 Fig — HL-1 cells were cultured at least during 3 weeks with normal (5.5 mmol/l) glucose then submitted to 4 different regimens (LG, NG, HG or IHG) for either 12 (A) or 72h (B). The oxidized proteins were detected with the oxyblot assay. Total proteins were detected in stain free gels and oxidized proteins after derivatization by DNPH. Results were expressed as the ratio of oxidized proteins/total proteins. Data are the means ± S.E.M of 5 independent experiments. One-way ANOVA followed by Dunnett’s multiple comparisons test. *, P < 0.05, **, P < 0.01, vs LG treatment, $, P < 0.05, vs NG treatment. (PPTX) [file pone.0289475.s008.pptx]

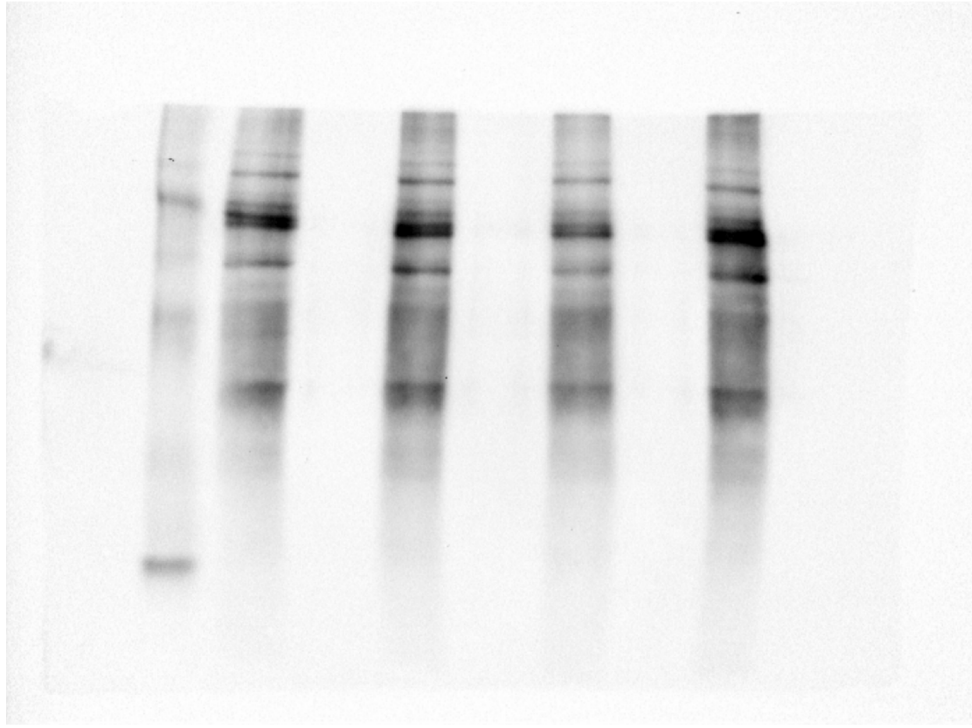

Oxyblot 12h treatment

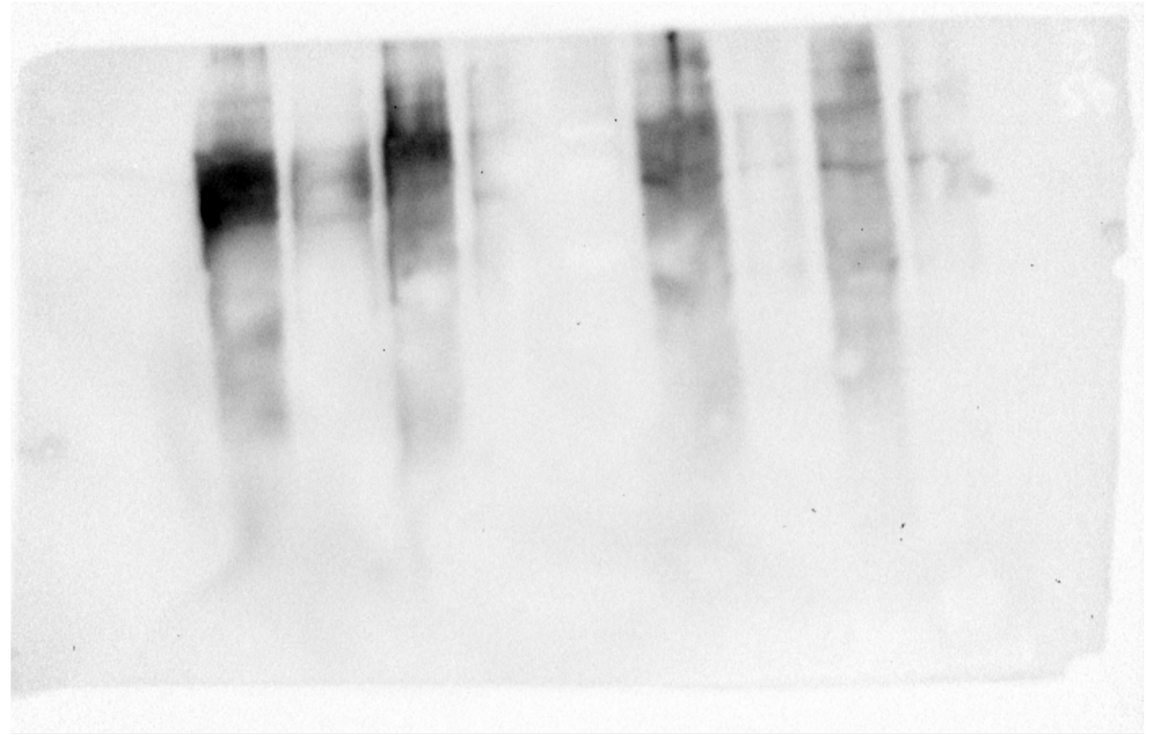

Oxyblot 72h treatment

Supplement: S1 Raw data — (PDF) [file pone.0289475.s009.pdf]
